# Mutation in CDC42 Gene Set as a Response Biomarker for Immune Checkpoint Inhibitor Therapy

**DOI:** 10.1002/cam4.70556

**Published:** 2025-01-10

**Authors:** Kun Wang, Yingying Zhang, Zhaoming Su, Bei Wang, Yuanyang Zhou, Xiaochu Tong, Chengying Xie, Xiaomin Luo, Sulin Zhang, Mingyue Zheng

**Affiliations:** ^1^ School of Life Sciences, Division of Life Sciences and Medicine University of Science and Technology of China Hefei China; ^2^ The First Affiliated Hospital of USTC (Anhui Provincial Hospital), division of Life Sciences and Medicine University of Science and Technology of China Hefei China; ^3^ Drug Discovery and Design Center, State Key Laboratory of Drug Research Shanghai Institute of Materia Medica, Chinese Academy of Sciences Shanghai China; ^4^ School of Chinese Materia Medica, Nanjing University of Chinese Medicine Nanjing China; ^5^ Shanghai Institute for Advanced Immunochemical Studies ShanghaiTech University Shanghai China

**Keywords:** biomarker, CDC42, clinical response, immunotherapy, pan‐cancer

## Abstract

**Background:**

Immune checkpoint inhibitors (ICIs) have achieved great success; however, a subset of patients exhibits no response. Consequently, there is a critical need for reliable predictive biomarkers. Our focus is on CDC42, which stimulates multiple signaling pathways promoting tumor growth. We hypothesize that an impaired function of CDC42 may serve as an indicator of a patient's response to ICI therapy.

**Methods:**

We consider CDC42 and its downstream binding and effector proteins as a gene set, as mutations in these components could lead to defective CDC42 function. To elucidate the biomarker function of mutations within the CDC42 gene set, we curated a comprehensive discovery dataset that included seven ICI treatment cohorts. And we curated two ICI treatment cohorts for validation. We explored the mechanism based on The Cancer Genome Atlas database. We also examined whether combining a CDC42 inhibitor with ICI could enhance ICI's efficacy.

**Results:**

Mutations in the CDC42 gene set were associated with improved overall survival and progression‐free survival. Furthermore, our analysis of immune response landscapes among different statuses of the CDC42 gene set supports its role as a biomarker. Animal experiments also revealed that the combination of the CDC42 inhibitor (ML141) with anti‐PD‐1 blockade can additively reduce tumor growth.

**Conclusions:**

Our study suggests that the CDC42 gene set mutations could potentially serve as a novel biomarker for the clinical response to ICI treatment. This finding also provides insights into the potential of combining ICI and CDC42 inhibitor use for more efficient patient treatment.

**Abbreviations**
CDC42: Cell division cycle 42
CXCL: C‐X‐C motif chemokine ligand
FDR: False Discovery rate
IL: Interleukin
MHC: major histocompatibility complex

## 1 Introduction

Immune inhibitor therapy, such as drugs targeting programmed cell death (ligand) 1 [PD‐(L)1] and cytotoxic T lymphocyte antigen 4 (CTLA‐4), has achieved great success in cancer therapy. However, the effectiveness of immune checkpoint inhibitor (ICI) drugs can vary greatly among different patients, attributed to tumor heterogeneity. While these drugs have shown positive effects in some patients, the majority do not exhibit clinical responses [1]. Therefore, identifying predictive biomarkers that can indicate clinical benefit for patients is crucial [2].

CDC42 is a type of ras homologous (rho) GTPase that stimulates tumor genesis, progression, invasion and metastatic [3]. In a previous study, it was reported that inhibiting CDC42 activity in regulatory T cells (Tregs) can enhance anti‐tumor immunity [4]. While Kalim et al. report that the immuno‐effect of the CD42 inhibitor outweighs any tumor cell‐intrinsic effect, it has also been reported that a low level CDC42 in the serum can predict the clinical response to ICI in patients with advanced hepatocellular carcinoma (HCC) [5] and advanced cervical cancer [6]. Therefore, it is valuable to explore the defective function of CDC42 signaling in tumors beyond Tregs and whether it can raise the probability of a response to ICI. If the defective function of CDC42 signaling is a biomarker for ICI therapy, it could provide further insight into the combined use of ICI and CDC42 inhibitor.

CDC42's role in controlling cell growth and polarity depends on both itself and its binding and effector proteins. Therefore, we consider CDC42, its binding protein, and effector protein as a gene set, investigating their potential as a biomarker to indicate the clinical benefit of ICI therapy. This involves exploring the potential biomarker role of defective CDC42 function indirectly. Using the ICI therapy discovery datasets and validation datasets, we examine whether a significant difference exists in overall survival (OS) and progression‐free survival (PFS) among patients with different statuses of the CDC42 gene set. To further elucidate the predictive performance of the CDC42 gene set status, we analyze the tumor intrinsic and tumor extrinsic immune response landscapes associated with its status.

Our study also aims to demonstrate how CDC42 inhibitors can potentially enhance the anti‐tumor effects of ICI therapy, particularly in cases where tumors exhibit resistance to ICI therapy. We selected mice with 4 T1 breast carcinoma, which has been shown to exhibit high resistance to anti‐PD‐1 or anti‐CTLA‐4 therapy [7] and selected ML141, a selective, non‐competitive inhibitor of CDC42 [8] for this experiment. Overall, our study explores the potential role of CDC42 gene set status as a biomarker for ICI therapy and seeks evidence supporting the use of a CDC42 inhibitor to enhance the efficacy of ICI therapy.

## 2 Materials and Methods

### 2.1 Patient Selection

We collected data from nine whole exome sequencing (WES) datasets of patients who underwent ICI therapy for biomarker discovery (Table S1). The Miao2019 cohort [9], the Hugo cohorts [10], the Riaz cohorts [11], the Miao2018 cohort [12], the Rizvi cohort [13], the Snyder cohorts [14], and the Van Allen cohorts [15], these seven datasets constituted the discovery cohort. The Hellmann cohort [16], and the Liu cohort [17], these two datasets were designated as the validation cohorts. We downloaded eight WES datasets and corresponding clinical information from the cBioPortal database (https://www.cbioportal.org). The Riaz cohort was obtained from the original literature [11].

In order to combine this data from multiple sources, we utilized the processing method described by Zhang et al. [18]. Initially, we excluded three tumor types with a sample size of less than 10. We also removed 33 samples that had a non‐evaluable response (NE), 7 samples that were not profiled and 7 samples classified as “OTHER CONCURRENT THERAPY”. Furthermore, we eliminated 151 duplicate samples in the Miao2018 cohort. This cohort had 27 overlapping samples with the Rizvi cohort, 37 with the Snyder cohort, and 87 with the Van Allen cohort. After filtering, only 10 patients with head and neck cancer were left, which were also deleted. Table 1 summarizes the characteristics of the filtered data. The ICI discovery cohort includes four tumor types and the following types of drug treatment: anti‐PD‐1, anti‐CTLA‐4 and anti‐CTLA‐4 + anti‐PD‐1. The validation cohort included melanoma and NSCLC, the two tumor types with the highest proportion in the discovery cohort (70% and 15%).

**TABLE 1:** Characteristics of the discovery cohort and validation cohort.

Characteristic	Num (Portion)	Validation cohort 1 (Hellmann cohort)	Validation cohort 2 (Liu cohort)
	Discovery cohort		
Gender			
Male	211 (52%)	32 (47%)	82 (59%)
Female	128 (32%)	36 (53%)	58 (41%)
NA	65 (16%)	—	—
Age			
< 65	160 (40%)	33 (49%)	—
≥ 65	105 (26%)	35 (51%)	—
NA	139 (34%)	—	140 (100%)
Cancer type			
Melanoma	282 (70%)	—	140 (100%)
Non‐small cell lung cancer	60 (15%)	68 (100%)	—
Renal cell carcinoma	35 (9%)	—	—
Bladder cancer	27 (6%)	—	—
Drug target			
Anti‐PD‐1	160 (40%)	—	140 (100%)
Anti‐CTLA‐4	174 (43%)	—	—
Anti‐CTLA‐4 + anti‐PD‐1	70 (17%)	68 (100%)	—
Treatment best response			
PR	77 (19%)	20 (29%)	38 (27%)
CR	19 (4%)	4 (6%)	17 (12%)
PD	172 (43%)	17 (25%)	65 (47%)
SD	81 (20%)	27 (40%)	20 (14%)
NA	55 (14%)	—	—
Durable clinical benefit			
Benefit	128 (32%)	37 (55%)	72 (51%)
Nonbenefit	201 (50%)	28 (41%)	68 (49%)
NA	75 (18%)	3 (4%)	
CDC42 gene set status			
Mutant	65 (16%)	22 (32%)	25 (18%)
Wild type	339 (84%)	46 (68%)	115 (82%)
Overall patients	404	68	140

Furthermore, we collected data from 32 types of solid cancer data from The Cancer Genome Atlas (TCGA) to conduct further analysis on the CDC42 gene set mutations as a biomarker. This data includes WES data and RNA‐seq data, which were obtained using TCGAbiolinks [19]. Additionally, we obtained Cibersort immune infiltration values and TCR Shannon for each TCGA cancer sample from Thorsson et al. [20].

### 2.2 CDC42 Gene Set Mutation Definition

We identified the CDC42 gene set, comprising key genes like CDC42, CDC42BPA, CDC42BPB, CDC42BPG (CDC42 binding protein kinase alpha, beta, gamma), CDC42EP1–5 (CDC42 effector protein 1–5), CDC42SE1–2 (CDC42 small effector 1–2). These genes are integral to CDC42 function, and any non‐synonymous mutation in this set signifies a mutated CDC42 gene set status. Such mutations could influence CDC42 signaling, potentially compromising its function.

### 2.3 Clinical Endpoint Analysis

The objective response rate (ORR) was defined as the proportion of patients who received ICI therapy and achieved a complete response (CR) or partial response (PR) [21]. Durable clinical benefit (DCB) was defined as a CR, PR, or stable diseases (SD) that lasted for more than 6 months [22].

### 2.4 Immune Cell Fraction Analysis

We obtained the leukocyte fraction from Thorsson et al. [20]. The lymphocyte fractions were aggregated by using the cibersort estimate, including B cells naïve, B cells memory, T cells CD4 naïve, T cells CD4 memory resting, T cells CD4 memory activated, T cells follicular helper, Tregs, T cells gamma delta, T cells CD8, NK cells resting, NK cells activated, and Plasma cells [20]. The molecular estimate for tumor‐infiltrating lymphocyte (TIL) fraction was obtained by multiplying the aggregated lymphocyte fraction from the cibersort estimate with the leukocyte fraction obtained from Thorsson et al. The image estimate for TIL fraction was obtained from Saltz et al. [23].

### 2.5 Immune Signatures Analysis

We obtained 29 immune signatures from He et al. [24] and performed single‐sample gene set enrichment analysis (ssGSEA) using the “GSVA” R package [25] based on these signatures.

### 2.6 Gene Set Enrichment Analysis (GSEA) Analysis

We used TCGA RNA‐seq data and the DESeq2 package [26] to identify differentially expressed genes. Subsequently, we conducted GSEA analysis on the Kyoto Encyclopedia of Genes and Genomes (KEGG) pathway using the clusterProfiler package [27].

### 2.7 Estimation of Cytolytic Activity

We estimated cytolytic activity (CYT) based on the method described by Rooney et al. [28]. This involves calculating the geometric mean of granzyme A (GZMA) and perforin 1 (PRF1) expression.

### 2.8 Mutation and Neoantigens Analysis

For this study, we assessed tumor mutational burden (TMB) using the number of non‐synonymous mutations. The data for nonsilent mutation, silent mutation, single nucleotide variation (SNV) neoantigens and indel neoantigens were obtained from Thorsson et al. [20].

### 2.9 Statistical Analysis

We used the two‐sided Fisher's exact test to explore the clinical benefit difference between CDC42 gene set status. The two‐sided Wilcoxon rank sum test was used to compare the TMB and neoantigen load (NAL) of ICI therapy data. We also used the two‐sided Wilcoxon rank sum test to compare TCGA gene expression levels, mutation rate, neoantigens, cell fraction, immune signatures, TCR Shannon and CYT between the CDC42 gene set mutation group and the CDC42 gene set wild type group. Additionally, we plotted the KM curve of PFS and OS using the logrank test based on CDC42 gene set status, which used the $\chi^{2}$ test statistic to calculate P values [29]. Fisher's exact test was implemented using the python package scipy [30]. The logrank test, Cox Proportional‐Hazards analysis, and Wilcoxon rank sum test were implemented using the survminer package and ggsignif package in R version 4.2.3 (https://www.r‐project.org). In in vivo pharmacodynamic study, statistical analysis was done by GraphPad software, version 9, and one‐way ANOVA followed by Bonferroni post hoc test was used to analyze multiple groups.

### 2.10 Animal Experiment

All procedures performed on animals were conducted in accordance with the Institutional Animal Care and Use Committee at the Shanghai Institute of Materia Medica, Chinese Academy of Sciences (IACUC Issue NO. 2023‐10‐ZMY‐03). For the pharmacodynamics experiment, BALB/c mice (6–8 weeks old) were purchased and inoculated subcutaneously with 1 × 10^6^ 4 T1 tumor cells into the right side of the mice's axilla. The animals were divided into four groups irregularly once the tumor volume reached approximately to 100 mm^3^. ML141 (#HY‐12755, MedChemExpress) was administered in a solution containing PEG300, dimethyl sulfoxide, and PBS [40/5/55 (v/v/v)]. The mice were then treated intraperitoneally with or without ML141 (30 mg/kg once a day) and/or 150 μg/mouse of anti‐PD‐1 antibody (#BE0273, BioXCell) every other day for one injection. Tumor volumes were calculated using the formula: V = (length×width^2^)/2. Body weights and tumor volumes of the mice were measured daily. The tumor growth inhibition (TGI) value was calculated using the formula: TGI = [1‐Relative Tumor Volume (Treatment)/Relative Tumor Volume (Vehicle)] × 100%.

### 2.11 Flow Cytometry Analysis

The tumor tissues were firstly digested into single cells using a digestion solution containing 0.001% hyaluronidase, 0.1% collagenase, 0.002% DNase, 120 μM MgCl_2_, and 120 μM CaCl_2_ in RPMI 1640 medium. Subsequently, red blood cells were lysed using ammonium chloride for 3 min and then the cell samples were stained with Fixable Viability Stain 510 (#564406, BD). The Fc receptors were blocked with TruStain FcX (anti‐mouse CD16/32) antibody (#101320, Biolegend) and stained with the following antibodies: APC‐Cy7 rat anti‐mouse CD45 (#557659, BD), FITC CD3 monoclonal antibody (17A2) (#11–0032‐82, Invitrogen), APC anti‐mouse CD25 (#17‐0251‐82, Invitrogen), BV786 rat‐anti‐mouse CD4 (#563331, BD), PE rat anti‐mouse FOXP3 (#563101, BD), and Brilliant Violet 421 anti‐mouse CD8a antibody (#100738, Biolegend). The stained cells were analyzed using the Agilent Novocyte 3000 instrument, and all data were analyzed with FlowJo software. Flow cytometry gating strategies were illustrated in Figure S1.

### 2.12 RT‐PCR Analysis

Total RNA was extracted from samples using the Vazyme FastPure Cell/Tissue Total RNA Isolation Kit (RC112‐01). RNA was reverse transcribed into cDNA using the HiScript III qRT SuperMix (Vazyme, R323‐01‐AC). RT‐qPCR was performed on the QuanStudio 5 PCR Detection System (Thermo Fisher) using the ChamQ SYBR qPCR Master Mix (Vazyme, Q331‐AA). All experiments were conducted according to the manufacturer's instructions (Vazyme Biotech). The primer sequences used are as follows: mouse Actb forward: tgagctgcgttttacaccct, mouse Actb reverse: gccttcaccgttccagtttt; mouse Arg‐1 forward: acattggcttgcgagacgta, mouse Arg‐1 reverse: atcaccttgccaatccccag; mouse CD206 forward: gactgctgctgagtccagtt, mouse CD206 reverse: agggatcgcctgttttccag; mouse IL‐10 forward: gctccaagaccaaggtgtct, mouse IL‐10 reverse: cggagagaggtacaaacgagg; mouse Nos2 forward: gttctcagcccaacaatacaaga, mouse Nos2 reverse: gtggacgggtcgatgtcac, mouse TNFα forward: aggctgccccgactacgt, mouse TNFα reverse: gactttctcctggtatgagatagcaaa; mouse IL‐1β forward: tcgctcagggtcacaagaaa, mouse IL‐1β reverse: catcagaggcaaggaggaaaa.

### 2.13 Histological Analyses

The tumor tissue samples from mice in each group were fixed in 4% formaldehyde solution and then sent to Wuhan Servicebio Technology Co. Ltd. for immunohistochemical staining. The images of these stained samples were viewed and captured using SlideViewer software, and the immunohistochemistry (IHC) results were statistically processed using ImageJ software.

## 3 Results

### 3.1 Mutation in CDC42 Gene Set Was Associated With Improved Clinical Outcomes for ICI Therapy

**FIGURE 1:**
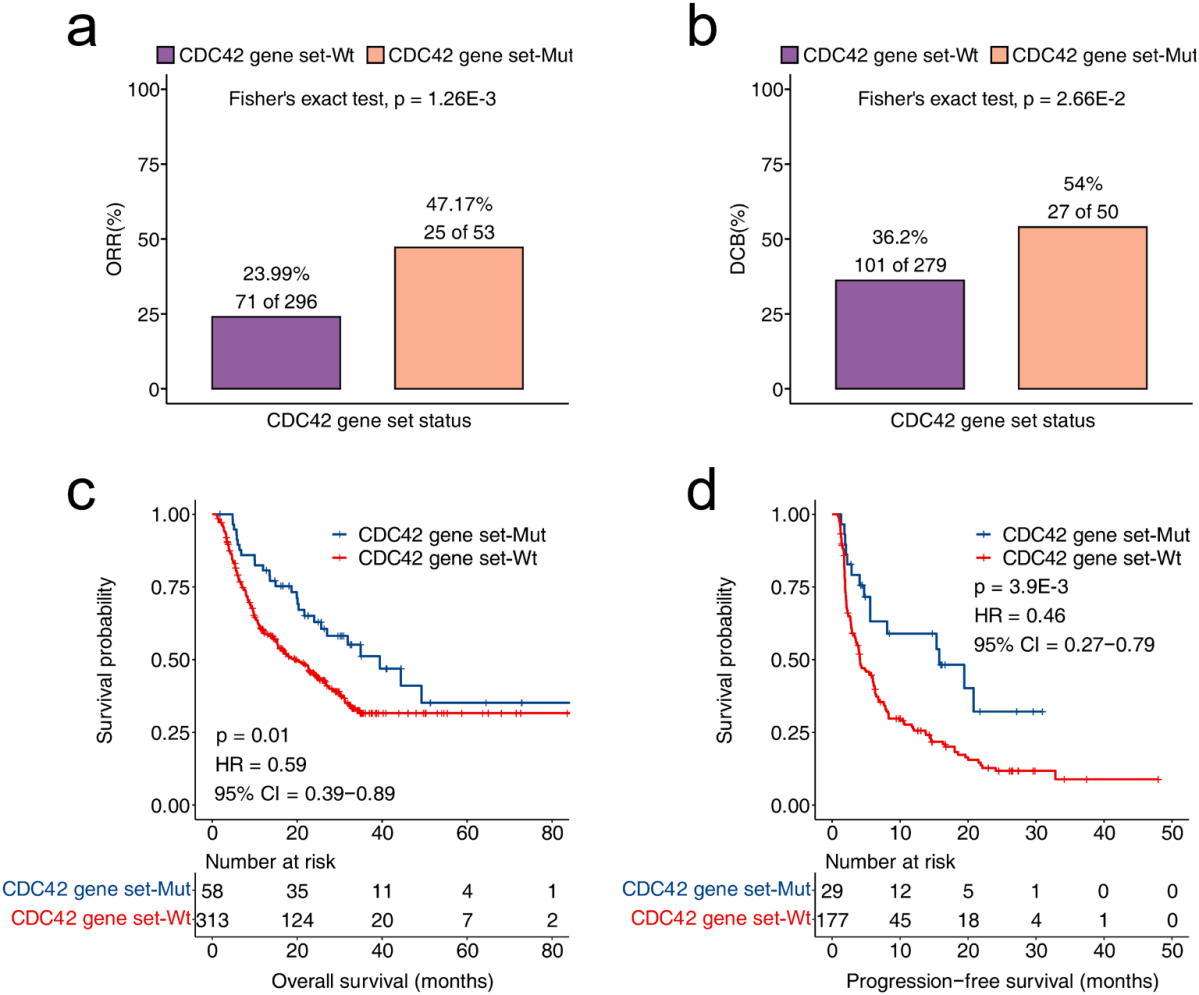
Analysis of CDC42 gene set mutation as a biomarker for ICI therapy in discovery cohort. The differences in (a) ORR, (b) DCB, between different CDC42 gene set statuses. The KM curve of (c). OS, (d) PFS, based on the CDC42 gene set status.

The results of discovery cohort were shown in Figure 1, the ORR of patients in the CDC42 gene set mutation was significantly higher (*p* = 1.26E‐3) compared to the CDC42 gene set wild type. Additionally, the DCB of patients in the CDC42 gene set mutation was also significantly higher (*p* = 2.66E‐2) than the wild type group. Moreover, CDC42 gene set mutation patients had significantly longer overall survival time (*p* = 0.01, HR = 0.59, 95% CI = 0.39–0.89) and PFS time (*p* = 3.9E‐3, HR = 0.46, 95% CI = 0.27–0.79) compared to the CDC42 gene set wild type group.

**FIGURE 2:**
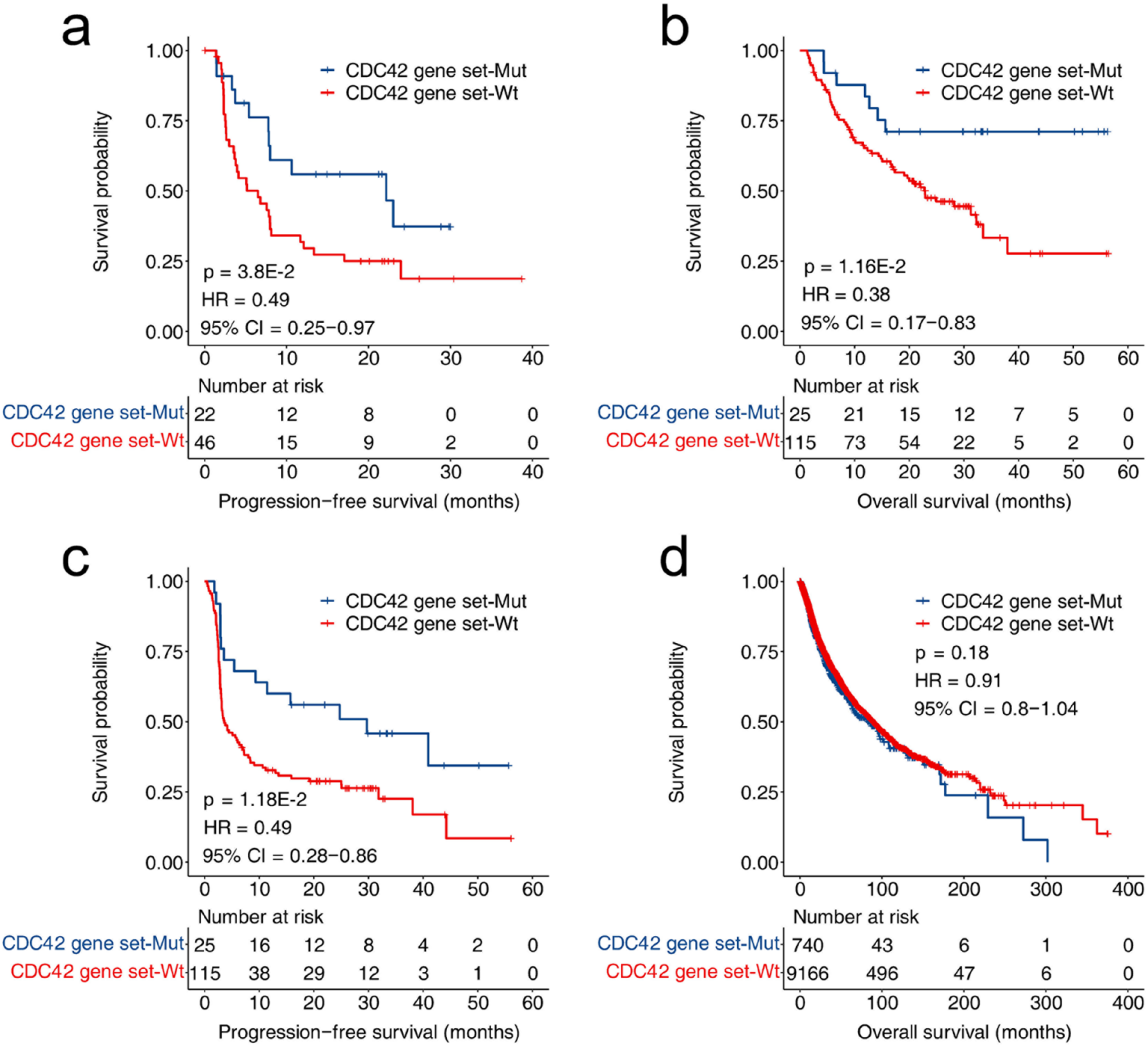
Analysis of CDC42 gene set mutation as a biomarker for ICI therapy in validation cohort. (a) The PFS KM curve of validation cohort 1 based on the CDC42 gene set status. (b) The OS KM curve of validation cohort 2 based on the CDC42 gene set status. (c) The PFS KM curve of validation cohort 2 based on the CDC42 gene set status. (d) The OS KM curve of TCGA cohort based on the CDC42 gene set status.

The biomarker function of CDC42 gene set mutations was validated on two validation cohorts. As shown in Figure 2a, the PFS of patients with CDC42 mutations in NSCLC patients was significantly longer than that of wild type patients (*p* = 3.8E‐2, HR = 0.49, 95% CI = 0.25–0.97). As shown in Figure 2b,c, in the melanoma validation cohort, patients with CDC42 gene set mutations had significantly longer PFS and OS (OS: *p* = 1.16E‐2, HR = 0.38, 95% CI = 0.17–0.83; PFS: *p* = 1.18E‐2, HR = 0.49, 95% CI = 0.28–0.86). Figure 2d shows the prediction of patient survival by CDC42 gene set in the TCGA dataset (*p* = 0.18, HR = 0.91, 95% CI = 0.8–1.04).

### 3.2 Assessment of Intrinsic Immune Response Landscapes in CDC42 Gene Set Wild Type and Mutation Tumors

**FIGURE 3:**
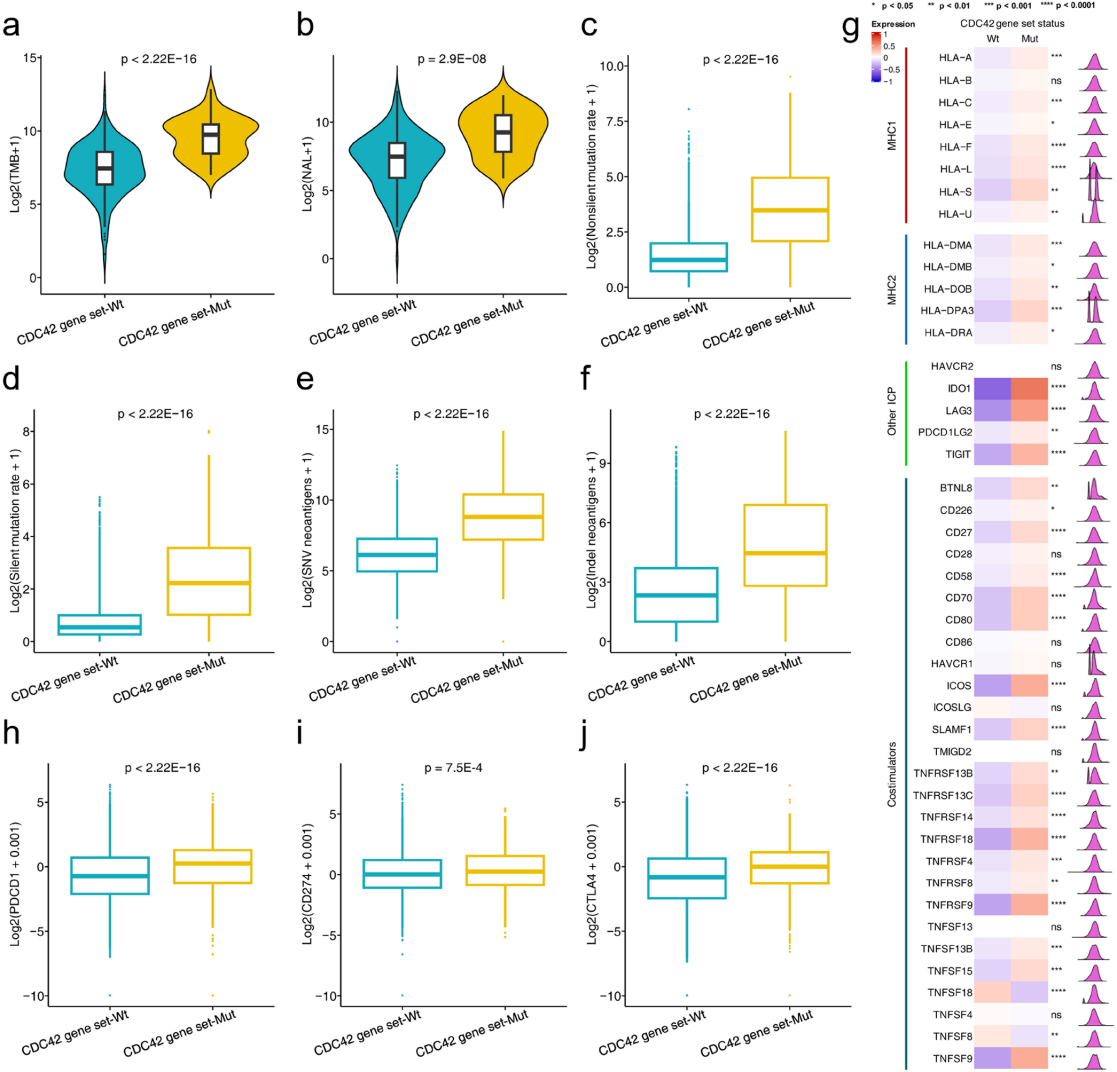
Analysis of intrinsic immune response landscapes between different CDC42 gene set statuses. Comparison of (a) TMB, (b) NAL between different CDC42 gene set statuses in the discovery cohort. Comparison of (c) nonsilent mutation rate, (d) silent mutation rate, (e) SNV neoantigens, (f) indel neoantigens, (g) expression of MHC and other ICP molecules and costimulators, (h) expression of PDCD1, (i) expression of CD274, (j) expression of CTLA‐4 between different CDC42 gene set statuses in the TCGA cohort. *p < 0.05, **p < 0.01, ***p < 0.001, ****p < 0.0001.

We initially examined the relationship between CDC42 gene set status and immunogenicity in the ICI therapy cohort. As shown in Figure 3a,b, the levels of TMB and NAL in the CDC42 gene set mutation group were significantly higher than those in the CDC42 gene set wild type group (TMB: *p* < 2.22E‐16, NAL: *p* = 2.9E‐8). These findings suggest increased immunogenicity in tumors with CDC42 gene set mutations. We also explored the relationship between CDC42 gene set status and immunogenicity in the TCGA cohort. Compared to CDC42 gene set wild type tumors, both the nonsilent mutation rate and the silent mutation rate were significantly higher in CDC42 gene set mutation tumors (*p* < 2.22E‐16, Figure 3c,d). Additionally, both SNV neoantigens and indel neoantigens were significantly more abundant in in CDC42 gene set mutation tumors compared to CDC42 gene set wild type tumors (*p* < 2.22E‐16, Figure 3e,f). These results in the TCGA cohort further support the notion that the CDC42 gene set mutations are associated with enhanced tumor immunogenicity.

Then, we investigated the relationship between CDC42 gene set status and the expression of immune‐related molecules, including two class MHC molecules, immune checkpoints, and co‐stimulators. We found that immune checkpoint genes PDCD1, CD274 and CTLA‐4 were upregulated in the CDC42 gene set mutation group, as shown in Figure 3h–j. Additionally, we observed significantly higher expression of MHC1, MHC2, other immune checkpoints (ICPs), and co‐stimulators in CDC42 gene set mutation tumors compared to CDC42 gene set wild type, as shown in Figure 3g.

### 3.3 Assessment Extrinsic Immune Response Landscapes in CDC42 Gene Set Wild Type and Mutation Tumors

The different situations of immune cell infiltration result in different clinical outcomes of ICI therapy [31]. Therefore, we investigated the difference in tumor microenvironment (TME) between CDC42 gene set wild type and mutation tumors. This included analyzing immune cell score, signatures representing immune cell function, and differential gene expression related to immune cell and ICI therapy efficiency.

**FIGURE 4:**
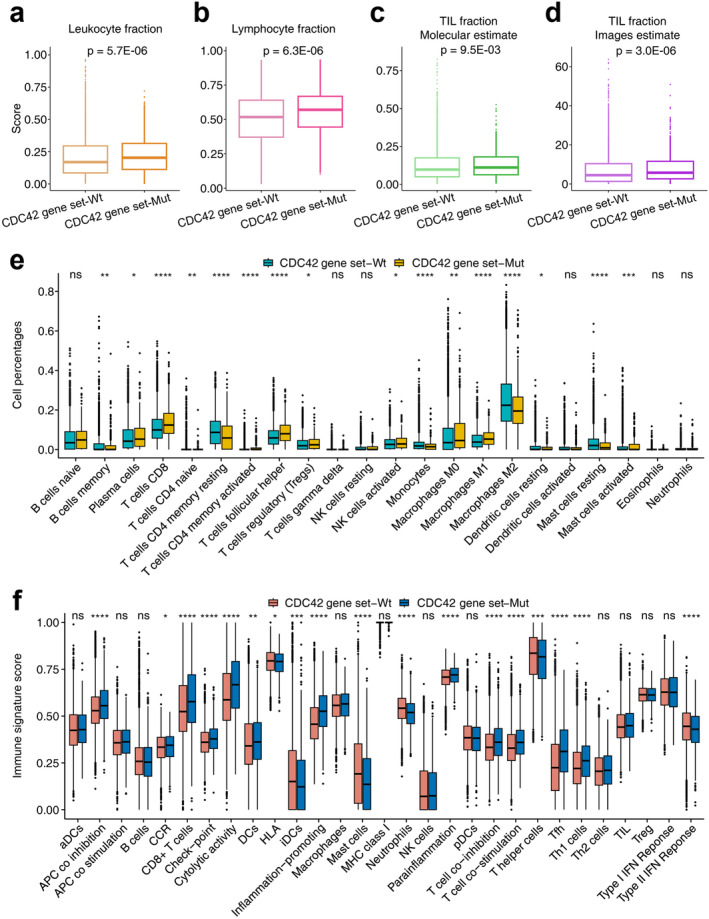
Analysis of extrinsic immune response landscapes of different CDC42 gene set statuses in TCGA cohort. Comparison of (a) leukocyte fraction, (b) lymphocyte fraction, (c) TIL fraction based on molecular estimates, (d) TIL fraction based on images estimates, (e) immune cell infiltration, (f) 29 immune signatures estimated through the ssGSEA method between different CDC42 gene set statuses. *p < 0.05, **p < 0.01, ***p < 0.001, ****p < 0.0001.

As shown in Figure 4a,b, the leukocyte fraction and lymphocyte fraction in CDC42 gene set mutation tumors were significantly higher than those in CDC42 wild type gene set tumors (leukocyte fraction, *p* = 5.7E‐06; lymphocyte fraction, *p* = 6.3E‐06). As TIL is crucial for killing tumors [32], TIL fractions estimated at both molecular and image levels were compared. Figure 4c shows that TIL fractions (molecular estimate) in CDC42 gene set mutation tumors are significantly higher than those in CDC42 gene set wild type tumors (*p* = 9.5E‐3). Figure 4d shows that TIL fractions (images estimate) in CDC42 gene set mutation tumors are significantly higher than in those CDC42 gene set wild type tumors (*p* = 3E‐06).

**FIGURE 5:**
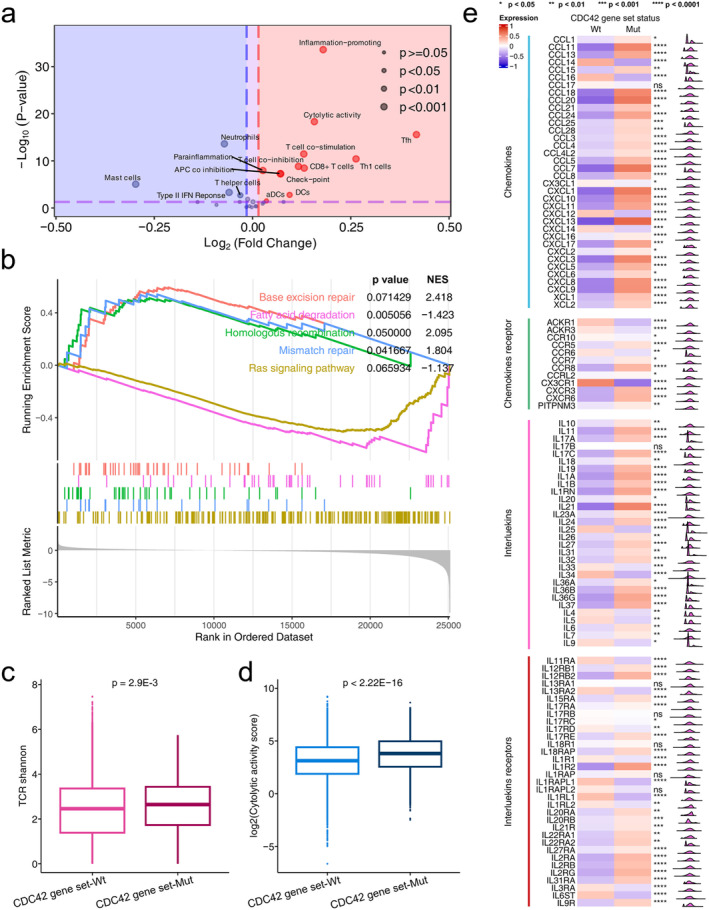
Analysis of gene expression related to immune cell between different CDC42 gene set statuses in TCGA cohort. (a) Volcano plots showing the analysis of 29 immune signatures estimated by the ssGSEA method for different CDC42 gene set statuses. (b) GSEA analysis (FDR <  0.25) results based on CDC42 gene set status. Comparison of the (c) TCR Shannon (d) cytolytic activity score, (e) chemokines (and receptors) and interleukins (and receptors) between different CDC42 gene set statuses. *p < 0.05, **p < 0.01, ***p < 0.001, ****p < 0.0001.

Cibersort scores based on the TCGA cohort were also compared between CDC42 gene set wild type and mutation tumors. As shown in Figure 4e, the percentages of immune cell types were compared in detail. We found significant differences in most of the immune cell scores between CDC42 gene set wild type and mutation tumors. For example, the CD8 T cell and macrophage M1 cell scores in the CDC42 gene set mutation type were significantly higher than that in the CDC42 gene set wild type tumors. Figure 4f shows ssGSEA results based on 29 immune signatures. We found that CD8 T cell and checkpoint signatures in CDC42 gene set mutation type tumors were significantly higher than those in CDC42 gene set wild type tumors. Figure 5a further shows that immune signatures were significantly enriched in CDC42 gene set mutation tumors compared to CDC42 gene set wild type tumors.

We further conducted GSEA analysis, as well as expression analysis of chemokines and chemokines receptors, interleukins and interleukins receptors, TCR, and cytolytic activity score based on CDC42 gene set status. In Figure 5b, we observed enrichment of base excision repair, homologous recombination, mismatch repair pathway enriched in CDC42 gene set mutation tumors, while fatty acid degradation and Ras signaling pathway were enriched in CDC42 gene set wild type tumors. These results related to ICI therapy response. For example, Jiang et al. reported that DDR pathways are associated with the response to ICIs treatment [33]. Ward et al. reported that activation of the Ras signaling pathway leads to an immunosuppressive tumor microenvironment, hindering T cells activation and infiltration, thus affecting the therapeutic efficacy of ICI [34]. Li et al. reported that increased lipid content is correlated with a favorable ICI therapy response [35], suggesting that higher lipid accumulation may indicate a higher likelihood of a positive response to ICI therapy. In Figure 5c, we found a higher TCR Shannon score in CDC42 gene set mutation tumors compared to that in CDC42 gene set wild tumors (*p* = 2.9E‐3). The cytolytic activity score in CDC42 gene set mutation patients was significantly higher than in CDC42 gene set wild type patients (Figure 5d,p < 2.22E‐16). As shown in Figure 5e, lots of chemokines in CDC42 gene set mutation patients were significantly higher than in CDC42 gene set wild type patients, such as CXCL9, CXCL10, CXCL11 and CXCL13. There were also significant differences in interleukins and interleukins receptors expression between CDC42 gene set statuses, such as IL‐10 and IL21. All these results could help prove CDC42 gene set mutations as effective biomarkers, see discussion.

### 3.4 Exploring CDC42 Inhibitor Combined With ICI to Enhance the Efficacy of Immunotherapy

**FIGURE 6:**
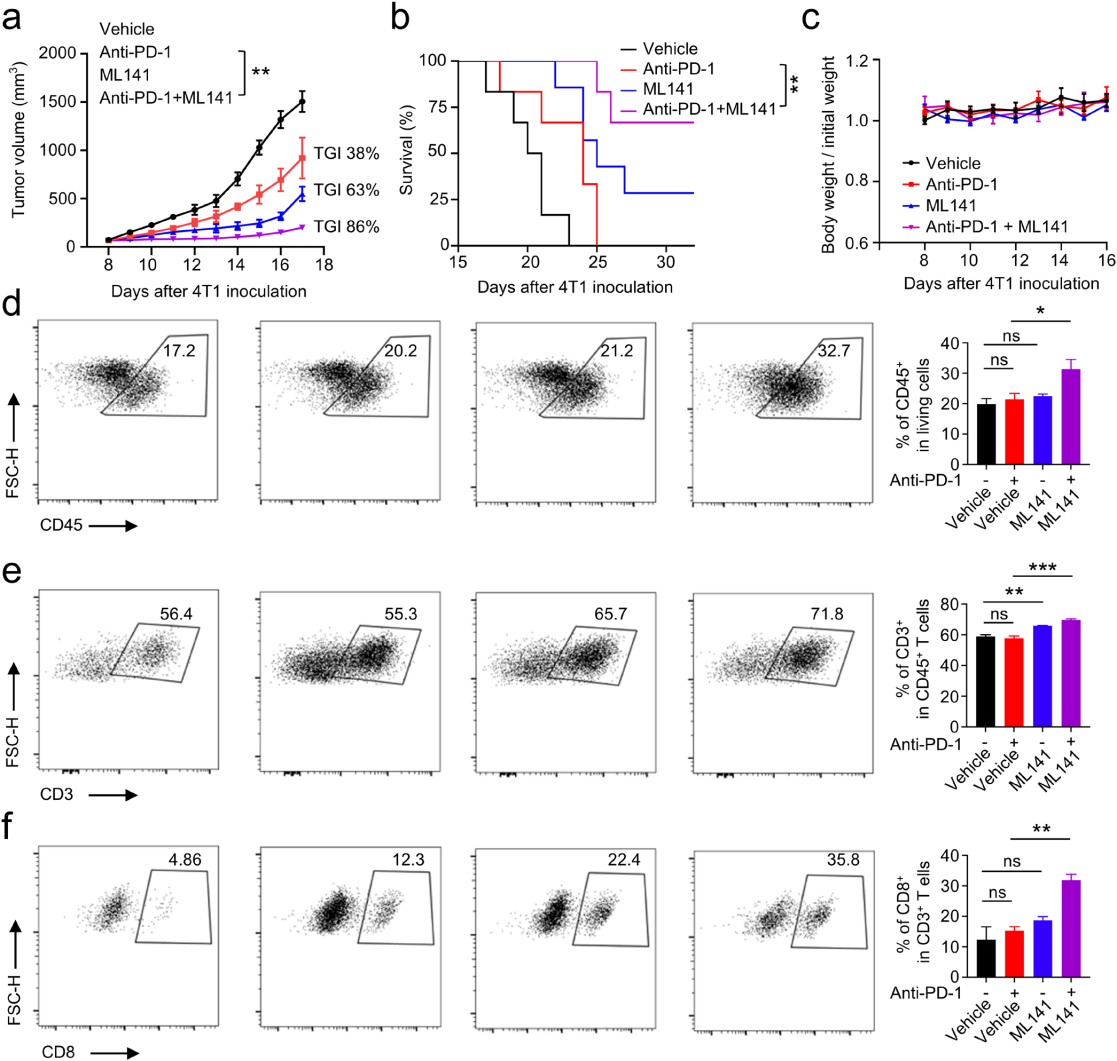
ML141 enhances Anti‐PD‐1 antibody induced tumor inhibition in vivo. (a) Growth curves of tumors from the indicated groups (*n* = 8). (b) Kaplan–Meier survival curves of mice in the indicated group (*n* = 6). (c) Body weight change curves of mice in the indicated group. (d–f) Impact of Anti‐PD‐1 antibody alone, ML141 alone, or a combination of both on the frequency of tumor‐infiltrating lymphocytes, CD3^+^ T cells, and CD8^+^ T cells in TEM, assessed by flow cytometry. Error bars represent mean ± SEM; statistical analysis was performed using one‐way ANOVA followed by Bonferroni post hoc test (a, d–f) or logrank test (b). ns, not significant; *, *p* < 0.05; **, *p* < 0.01; ***p < 0.001.

We investigated the antitumor effect of combining a CDC42 inhibitor (ML141) with anti‐PD‐1 antibody. Treatment with anti‐PD‐1 antibody alone demonstrated ordinary therapeutic capacity, with a TGI of 38% (Figure 6a). Treatment with ML141 alone resulted in a TGI of 63% (Figure 6a). Notably, the combination of ML141 and anti‐PD‐1 antibody significantly reduced tumor growth (TGI = 86%) and prolonged survival time compared to the anti‐PD‐1 antibody alone group (Figure 6a,b). This suggests that the combination therapy can effectively exert potent antitumor immune activity. Furthermore, these treatments did not lead to weight loss in the mice (Figure 6c), indicating that this dosing regimen is safe. To determine the role of the immune response in the antitumor activity of this dosage regimen, we analyzed the immune cells' infiltration in the TME using flow cytometry experiments. The results showed that a combination of ML141 and an anti‐PD‐1 antibody significantly increased the frequency of CD45^+^ lymphocytes, CD3^+^ T cells, and CD8^+^ cytotoxic T cells in the TME compared to the anti‐PD‐1 antibody alone (Figure 6d–f). However, it does not affect the frequency of CD4^+^CD25^+^FOXP3^+^ regulatory T cells (Tregs) in the TME (Figure S2). To further analyze the distribution and quantity of CD3^+^ T cells and CD8^+^ cytotoxic T cells in tumor tissue, we performed IHC staining. The results showed that the distribution ratio of CD3^+^ and CD8^+^ cells was significantly higher in the combination therapy group compared to the monotherapy group (Figure S3). The use of ML141 alone also raised the frequency of CD3^+^ T cells in the TME, aligning with earlier discoveries that the pharmacological inhibition of CDC42 triggers antitumor immune activity [4]. We also analyzed the expression of M1 markers (IL‐1β, Nos2, TNFα) and M2 markers (Arg‐1, CD206, IL‐10) in tumor‐associated macrophages. The results showed that, compared to the anti‐PD‐1 antibody alone, the combination of ML141 and the anti‐PD‐1 antibody significantly increased the expression of M1 markers and significantly reduced the expression of M2 markers in tumor‐associated macrophages (Figure S4). This suggests that the combination therapy can significantly promote the transformation of tumor‐associated macrophages into the M1 type, compared to the monotherapy. In conclusion, these findings suggest that the use of a CDC42 inhibitor in conjunction with an ICI has an additive therapeutic effect, enhancing the efficacy of immunotherapy.

## 4 Discussion

CDC42 downstream signals play a role in promoting various aspects of tumor development, including tumorigenesis, progression, invasion, and metastasis [3]. Our hypothesis posits that CDC42's functionality depends on both itself and downstream binding proteins and effector functions, collectively termed the CDC42 gene set. Mutations in these genes may result in a partial defect in CDC42 function. Analyzing the discovery cohort revealed that patients with mutations in the CDC42 gene set exhibited a higher ORR and DCB. Furthermore, patients with CDC42 gene set mutations experienced significantly extended OS and PFS compared to those with CDC42 gene set wild type in the discovery cohort and validation cohort. And CDC42 gene set mutations were not prognostic predictors. These analyses suggest that CDC42 gene set mutations could serve as a clinical biomarker for ICI therapy.

We conducted a further analysis of the CDC42 gene set's status to better understand its role in indicating the clinical response to ICI, using the ICI therapy cohorts and the TCGA dataset. Our findings revealed that tumors with CDC42 gene set mutations exhibited stronger immunogenicity, as evidenced by higher TMB and NAL. The increased immunogenicity indicates a higher likelihood of positive responses to ICI therapy and are more likely to be recognized and killed by immune cells than CDC42 gene set wild type tumors. Additionally, we compared the gene expression levels/scores of immune checkpoint‐related genes, MHC1, MHC2, Costimulators, TCR Shannon, and CYT, and observed significantly higher expression levels/scores in patients with CDC42 gene set mutations. Previous studies have suggested that higher expression of immune checkpoint‐related genes is indicative of a better response to ICIs therapy [36, 37]. MHC1 plays a crucial role in presenting antigens to CD8 T cells, and its down‐regulation is associated with resistance to ICIs [38]. Moreover, positive expression of MHC2 correlates with a response to ICIs therapy [39]. Costimulators can promote T cell activation and survival, and activation of co‐stimulatory pathways enhances checkpoint inhibition [40, 41], which could mean that patients with CDC42 gene set mutations are more suitable for ICI therapy. Higher TCR diversity may indicate that T cells can recognize more neoantigens, and studies have shown that patients with higher TCR diversity scores have more favorable clinical responses to ICI treatment [42]. CYT is upregulated during T cell activation [43], indicating that upregulated CYT leads to more effective tumor killing. Therefore, elevated level of TCR diversity and high expression of CYT indicate T cell activation and enhanced tumor cell killing efficiency. These comparisons highlight a more active immune response in CDC42 gene set mutation patients, characterized by stronger immunogenicity and the potential for more effective T cell activation and killing through increased MHC1 antigen presentation and TCR diversity, as well as higher CYT expression.

In TME, leukocyte fraction, lymphocyte fraction, and TIL fraction were substantially higher in patients with CDC42 gene set mutations compared to those with wild type, indicating enhanced immunity. These results indicate that CDC42 gene set mutation tumors are more likely to be recognized and killed by immune cells than CDC42 gene set wild type tumors. Analysis of specific immune cell types revealed that CD8 T cell levels and macrophage M1 substantially higher in patients with CDC42 gene set mutations. Immune signatures analysis also revealed that CD8 T cells and checkpoint signatures in CDC42 gene set mutation type tumors were significantly higher. These findings are consistent with previous reports that CD8 T cell are key determinants of response to ICI, and macrophage M1 cells are related to T cell stimulation and ICI therapy [37, 44]. Additionally, higher expression of immune checkpoint‐related genes is more likely to benefit clinically from ICIs treatment [36].

The increased expression of chemokines, such as CXCL9, CXCL10, CXCL11 and CXCL13, in patients with CDC42 gene set mutations can promote efficacy of ICI. Previous studies have reported that CXCL9, CXCL10 and CXCL11 can enhance T cell infiltration, thereby improving the therapeutic efficacy of ICI interventions [45, 46, 47]. The expression of CXCL13 can generate effector T cells and is closely associated with the response to ICIs treatment [48]. Additionally, higher expression of interleukins, like IL‐10 and IL‐21, in CDC42 gene set mutation patients promotes the survival of T cells. Pegilodecakin (PEGylated recombinant IL‐10) induces the proliferation of CD8 T cells both within the tumor microenvironment and in the systemic circulation, while also activating CD8 T cells within TME [49]. IL‐21 functions as a robust survival factor for both natural killer (NK) and T cells, while also inhibiting the differentiation of Tregs [50]. Therefore, the significantly high expression of IL‐10 and IL‐21 in CDC42 gene set mutation patients may indicate the presence of more CD8 T cells in TME and a higher probability of positive response to ICI therapy compared to patients with CDC42 gene set wild type status.

Mutations in the CDC42 gene set could lead to the defective function of CDC42 and the inhibition of tumor growth, which further alleviates immune suppression. Hence, we propose that the combined use of ICI and CDC42 inhibitors could potentially enhance ICI's efficacy, and we conducted experimental exploration, which demonstrated that the ML141 inhibitor could indeed further promote ICI's efficacy.

In summary, our exploration reveals that mutations of CDC42 gene set could serve as a biomarker of the clinical response to ICI therapy. Additionally, our study sheds light on the potential additive effects of combining CDC42 inhibitors with ICIs, offering promise, especially in cases that did not respond to anti‐PD‐1 treatment.

## Author Contributions

**Kun Wang:** conceptualization (equal), data curation (equal), investigation (equal), methodology (equal), visualization (equal), writing – original draft (equal), writing – review and editing (equal). **Yingying Zhang:** conceptualization (equal), data curation (equal), investigation (equal), methodology (equal), visualization (equal), writing – original draft (equal), writing – review and editing (equal). **Zhaoming Su:** data curation (supporting), methodology (supporting). **Bei Wang:** data curation (supporting), methodology (supporting). **Yuanyang Zhou:** data curation (supporting), methodology (supporting). **Xiaochu Tong:** methodology (supporting). **Chengying Xie:** supervision (supporting). **Xiaomin Luo:** supervision (supporting). **Sulin Zhang:** conceptualization (equal), investigation (equal), methodology (equal), project administration (equal), supervision (equal), writing – review and editing (equal). **Mingyue Zheng:** conceptualization (equal), funding acquisition (lead), investigation (equal), methodology (equal), project administration (equal), supervision (equal), writing – review and editing (equal).

## Consent

No written consent has been obtained from the patients as there is no patient identifiable data included.

## Conflicts of Interest

The authors declare no conflicts of interest.

## Approval of the Research Protocol by an Institutional Reviewer Board

Not applicable to humans since there are no human subjects or samples in this study. All procedures performed on animals were conducted in accordance with the Institutional Animal Care and Use Committee at the Shanghai Institute of Materia Medica, Chinese Academy of Sciences (IACUC Issue NO. 2023‐10‐ZMY‐03).

## Supporting information


Table S1.
**Figure S1**.
**Figure S2**.
**Figure S3**.
**Figure S4**.

## Data Availability

The datasets supporting the conclusions of this article are publicly available and included within the article.
