# Supplementary material for: Mutation in CDC42 Gene Set as a Response Biomarker for Immune Checkpoint Inhibitor Therapy
Source: Cancer Med. 2025 Jan 10;14(1):e70556. doi: 10.1002/cam4.70556 (PMC11719708; doi:10.1002/cam4.70556)
Supplement: Supplementary file 1 — Table S1. Figure S1. Figure S2. Figure S3. Figure S4. [file CAM4-14-e70556-s001.docx]

**Supporting Information**

Table of Contents

| 1. Supplementary Table 1  2. Supplementary Figure 1 |
| --- |
| 3. Supplementary Figure 2 |
| 4. Supplementary Figure 3 |
| 5. Supplementary Figure 4 |

| **Data sets** | **Target** | **Cancer type** | **Patient number** |
| --- | --- | --- | --- |
| Miao2019 cohort | Anti-PD-1 | Renal Clear Cell Carcinoma | 35 |
| Hugo cohort | Anti-PD-1 | Melanoma | 38 |
| Miao2018 cohort | Anti-CTLA-4  Anti-PD-1  Anti-CTLA-4 + Anti-PD-1 | Non-Small Cell Lung Cancer  Bladder Cancer  Melanoma  Head and Neck Cancer | 249 |
| Rizvi cohort | Anti-PD-1 | Non-Small Cell Lung Cancer | 35 |
| Snyder cohort | Anti-CTLA-4 | Melanoma | 64 |
| Van Allen cohort | Anti-CTLA-4 | Melanoma | 110 |
| Riaz cohort | Anti-PD-1 | Melanoma | 73 |
| Hellmann cohort | Anti-CTLA-4 + Anti-PD-1 | Non-Small Cell Lung Cancer | 75 |
| Liu cohort | Anti-PD-1 | Melanoma | 144 |

**1. Supplementary Table 1:**

**Description of WES Data sets that received ICI therapy.**

**2. Supplementary Figure 1**


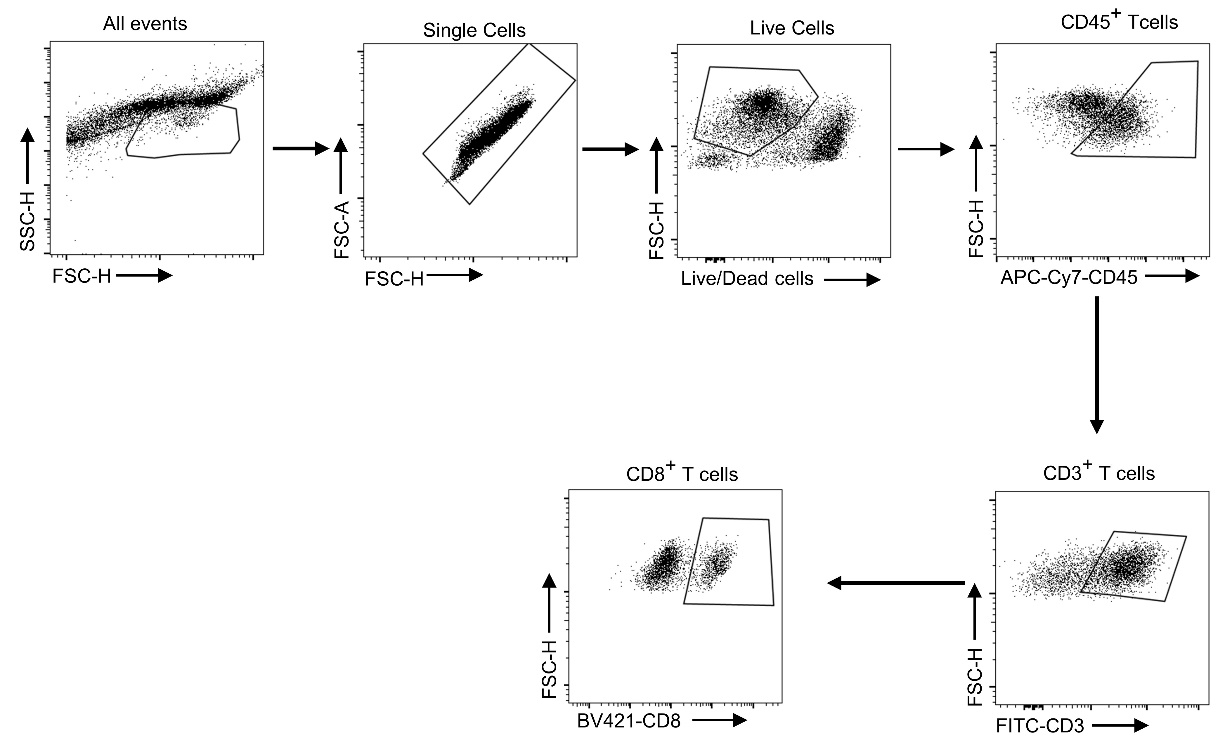


**Supplementary Figure 1**. Flow cytometry gating strategies.

**3. Supplementary Figure 2**


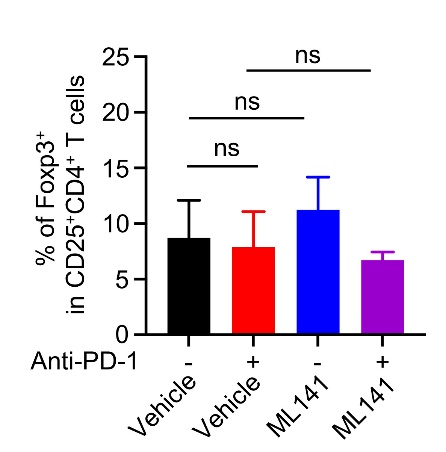


**Supplementary Figure 2**. **The expression of tumor-infiltrating CD4^+^CD25^+^FOXP3^+^ Tregs within the TME**. Statistical analysis was performed using one-way ANOVA followed by Bonferroni post hoc test. Data are represented as mean ± SEM. ns, not significant.

**4. Supplementary Figure 3**


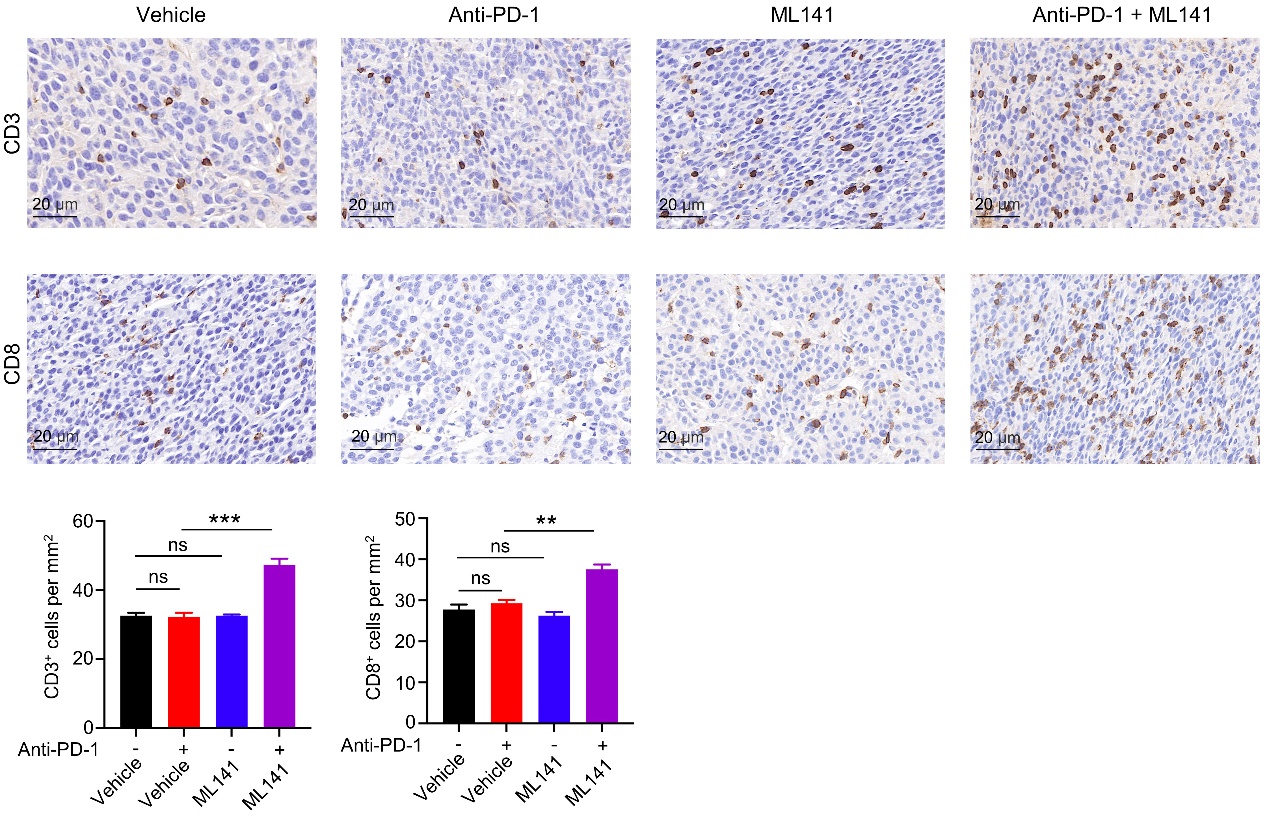


**Supplementary Figure 3. The distribution and quantity of CD3^+^ T cells and CD8^+^ cytotoxic T cells in tumor tissues.** The tumor tissues were obtained from each group and subsequently stained with antibodies against CD3 and CD8. The representative images were shown, scale bars represent 20 μm. Quantification analysis results were also shown. Statistical analysis was performed using one-way ANOVA followed by Bonferroni post hoc test. Data are represented as mean ± SEM. ns, not significant; **P<0.01; ***P<0.001.

**5. Supplementary Figure 4**


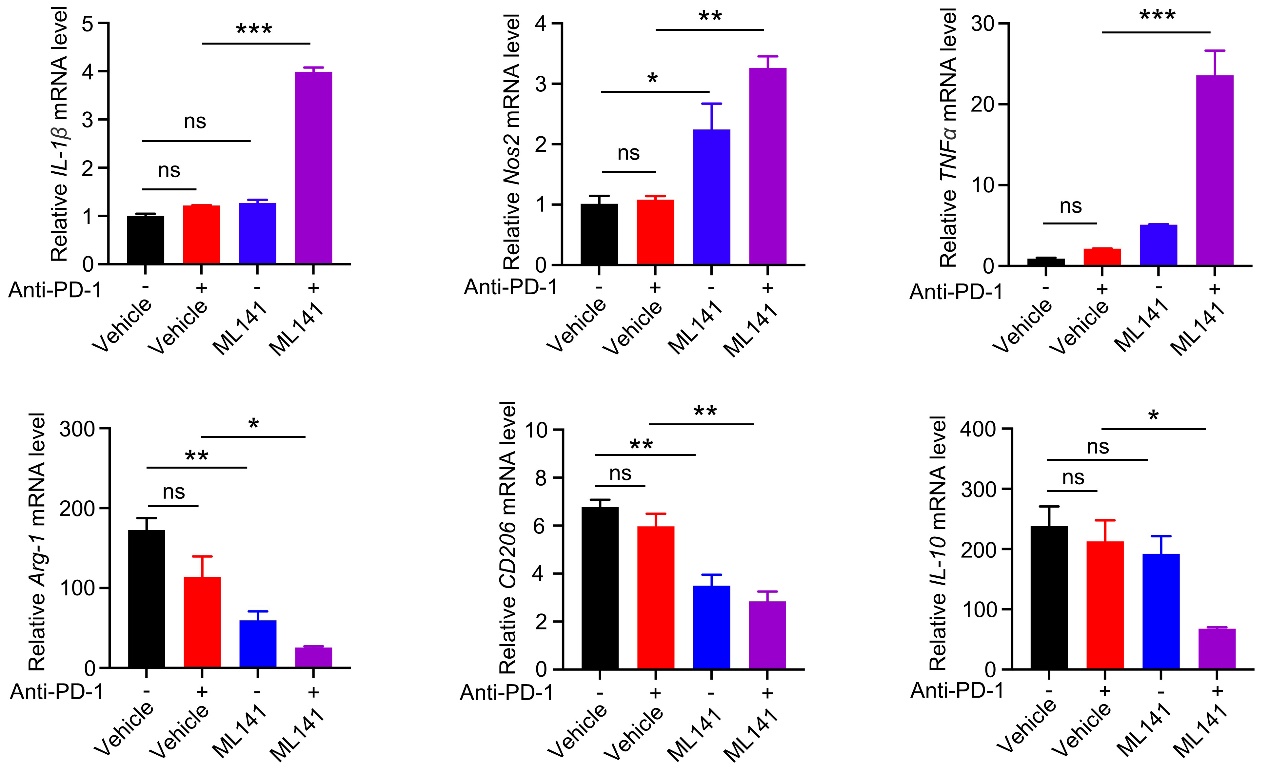
 **Supplementary Figure 4. The mRNA levels of M1 and M2 markers in tumor associated macrophage.** RT-qPCR was used to explore the impact of combined treatment with CDC42 inhibition and immunotherapy on macrophage polarization in 4T1 tumor microenvironment. At the end of the experiment, whole tumors were processed and tumor associated macrophage were isolated, the M1 macrophages marker (IL-1β, Nos2, TNFα) and M2 macrophages marker (Arg-1, CD206, IL-10) mRNA levels were measured by RT-qPCR. Statistical analysis was performed using one-way ANOVA followed by Bonferroni post hoc test. Data are represented as mean ± SEM. ns, not significant; *, P< 0.05; **P<0.01; ***P<0.001.
